# Depression and experience of incarceration in North Central Nigeria: a situation analysis at Makurdi medium security prison

**DOI:** 10.1186/s13033-020-00408-0

**Published:** 2020-10-27

**Authors:** Emeka Nwefoh, Chinyere M. Aguocha, Grace Ryan, Philip Ode, Festus O. Ighagbon, Oyedele Akinjola, Samuel Omoi, Jibril Abdulmalik, Terkura M. Agbir, Obekpa Obekpa, Samuel Ogbole, Julian Eaton

**Affiliations:** 1 CBM Country Co-Ordination Office, Abuja, Nigeria; 2 Department of Internal Medicine, Imo State University, Owerri, Nigeria; 3 Centre for Global Mental Health, London School of Hygiene and Tropical Medicine, London, UK; 4 Benue State University Teaching Hospital, Makurdi, Nigeria; 5 Benue State University, Makurdi, Nigeria; 6 Department of Psychiatry, University of Ibadan, Ibadan, Nigeria; 7 Federal Medical Center, Makurdi, Nigeria; 8 Benue State Comprehensive Community Mental Health Programme, Otukpo, Nigeria; 9 CBM Global and Centre for Global Mental Health, London School of Hygiene and Tropical Medicine, London, UK

**Keywords:** Depression, Prison, Experience of incarceration

## Abstract

**Background:**

Human rights watchdogs have described conditions in Nigerian correctional facilities and detention centers as damaging to the physical and mental health of inmates. While the prevalence of mental disorders is high, access to appropriate healthcare is grossly inadequate. Understanding the current state of prison inmates’ mental health and well-being is an essential first step to addressing this important issue. This study aims to document the mental health and experiences of incarceration of inmates of the largest medium security prison in Nigeria’s Benue State.

**Methods:**

A cross-sectional survey and descriptive analysis was carried out with a random sample of 381 prison inmates of Benue State Makurdi Medium Security Prison. Survey tools included: (1) a structured questionnaire on participants’ experiences in prison, and (2) the Patient Health Questionnaire (PHQ-9), a screening tool for depression.

**Results:**

Most participants were young men (95.5%, mean age 27.95) and had completed secondary school (63.5%). While prison authorities had identified only 27 participants as having a mental disorder, 144 (37.8%) screened positive for depression. Twenty six had received professional counseling while in prison. Of the six participants who were already taking a psychotropic medication at the time of imprisonment, four received medication after being imprisoned. Approximately half, (52%) of participants were dissatisfied with prison health care.

**Conclusions:**

Despite the high prevalence of depression among prison inmates, few cases are detected and treated. Prison staff may not recognize depression as a mental disorder, and the mental health care available is generally poor. Inadequate mental health and social care not only affects prison inmates’ well-being, but may also impact recidivism and health outcomes upon release. Prison inmates should be screened routinely for depression and other less-commonly recognized mental health conditions, and appropriate treatment made available.

## Background

In 2018, researchers from around the world launched a global call to action on the mental health of incarcerated people [1]. Noting the dearth of epidemiological and intervention studies on the mental health of prison inmates in low- and middle-income countries (LMICs), they proposed that evidence-based task-sharing interventions tested in low-resource community settings could be adapted for delivery in correctional facilities and post-release. An essential first step is to document the services currently available in LMIC prisons, the mental health of prison populations, and the stressors they face.

### Depression in prisons

#### Prevalence

Of the more than 10.74 million people imprisoned around the world [2], one in seven has a severe mental illness (schizophrenia and related disorders, and affective disorders including bipolar disorder and major depression) [3, 4]. A systematic review and meta-regression analysis of data from 24 countries calculated a 10.2% pooled prevalence of major depression among male prison inmates, and 14.1% among female prison inmates. The authors called for more studies from prison populations in low- and middle-income countries (LMICs) [3]. Lovett et al.’s subsequent meta-analysis of prevalence studies among prison inmates in Africa specifically estimates the pooled prevalence of mood disorders to be 22% [5]. This meta-analysis struggled with heterogeneity of classification, and the higher rate probably reflects a wider definition of ‘depression’, covering mild as well as moderate and severe depression. Mild to moderate depression, with anxiety disorders, are typically termed common mental disorders in the current literature, whilst major depression is classified as a severe mental illness.[3]. Several studies suggest that the prevalence of depression in Nigerian prison inmates exceed these regional and international estimates; however, most of this research comes from relatively prosperous cities in the southern part of the country. For example, a study carried out in Port Harcourt prison estimates that 37% of prisoners have depression [6]. Osasona and Koleoso report that nearly three quarters (72.6%) of inmates in their sample from a medium security correctional facility in Benin City show symptoms of depression [7]. In a sample from Ilesa correctional facility, 85.3% scored above the cut-off on the Depression Sub-scale of the Hospital Anxiety and Depression Scale (HADS) [8]. The only northern prevalence studies we have been able to identify were from the Plateau State capital, Jos [9, 10]. Here, they estimate the prevalence of depression among prison inmates at 30.8%, first using the General Health Questionnaire (GHQ-28) as a screening tool, followed by the Composite International Diagnostic Interview (CIDI) to confirm diagnosis.

While prevalence rates differ between studies, global systematic reviews indicate that the prevalence of depression among prison inmates is consistently higher than in the general population [3, 4]. This would certainly appear to be the case in Nigeria, where the Nigerian Survey of Mental Health and Well-being has estimated that only 12.1% of people will experience a diagnosable mental disorder (according to Diagnostic Statistical Manual [DSM] criteria) in their lifetime [11].

#### Causation

A general dearth of longitudinal studies makes it difficult to demonstrate the direction of causality for the association between mental disorders and imprisonment. However, a narrative synthesis of qualitative studies from high-income countries suggests that most prison inmates perceive the prison environment as having a negative impact on their mental health [12]. An exceptional few highlight the opportunity to access health services as a benefit of imprisonment. However, a general paucity of physical and mental health services (lack of human resources, timely mental health assessments and psychotropic medications) and psychosocial interventions (limited rehabilitation, vocational and community rehabilitation services) has been observed in prison studies across Africa [5]. They also report other worrying conditions that could represent significant psychosocial stressors to prisoners in low-resource settings: poor sanitation, lack of food and opportunities for recreation; poor communication between the medical and justice systems; and delays in trials, case-processing and release. It has long been acknowledged that the uncertain outcome of court hearings and the welfare of dependents during incarceration are important risk factors for mental disorders among prison inmates, and that speedy trials are necessary to avoid prolonged exposure to these and other stressors [13].

In Nigeria specifically, human rights watchdogs have attributed the high rate of mental disorders among prison inmates to overcrowding and lack of privacy, violence, enforced solitude, lack of meaningful activity, isolation from social networks, insecurity about future prospects and inadequate mental health services [14]. Several studies of the prevalence of mood disorders in Nigerian prisons have also identified associated factors [5], including: demographic characteristics (age, marital status, living situation prior to imprisonment) [6, 7]; medical history (current medical complaints, co-morbid physical illness, personal and familial history of mental disorder, self-reported metal health) [6–8, 15, 16]; frequency of visits to prisoners [17]; duration and status of imprisonment (prison term length, trial status) [7, 8]; and prison conditions and services (self-reported ratings of prison accommodation, feeding and healthcare) [7, 8].

#### Outcomes

Reviews of the global literature indicate that high rates of depression among prisoners can have significant consequences, both for prisoners and for the justice system more broadly [3, 4]. Prison inmates are at much higher risk of self-harm and suicide, which are often (though not always) linked to mental disorders—depression, in particular [4, 18]. In high-income countries, prison inmates with depression have 4.36 times higher risk of self-harm or suicide, compared to prison inmates with no known mental disorders [18]. Prison inmates with mental disorders are also more likely to experience violence and victimization (physical and sexual) from others while in prison [4]. A 2009 systematic review and meta-analysis concludes that people with psychotic disorders have 1.6 times the odds of repeat offending, compared to people with no mental disorders [19]. However, the odds are equivalent (OR 1.0) when psychotic disorders are compared with other mental disorders, indicating that people with mental disorders generally have an elevated risk of repeat offending.

#### Correctional facilities in Nigeria

While Nigeria’s first Western-style correctional facilities were created in 1861, the Nigerian Prisons Service was established with the post-Independence passage of national legislation in 1972 [20]. Recent estimates suggest the total number of prison inmates in Nigeria is around 72,000 [21, 22]. Reports have revealed that at least 65 percent of Nigeria's inmates have never been convicted of any crime, with some awaiting trial for up to 10 years [23]. Most cannot afford legal representation, and only one in seven of those awaiting trial have private legal representation [14].

Human rights watchdogs have described correctional facilities conditions in the country as harsh, appalling and damaging to the physical and mental health of inmates [14, 24]. Correctional facilities are overstretched and overcrowded, with many holding three times their designated capacities [21]. There are reports of Nigerian prison inmates sleeping two to a bed or on the floor in filthy cells, with poor sanitation and food and medication in short supply [14]. Prison inmates are routinely tortured, beaten and abused [24] and female inmates especially face the threat of rape [14, 24]. Inmates have also been denied contact with families and friends unless they can afford to bribe prison guards [14].

Medical treatment of Nigerian prison inmates is grossly inadequate [24]. It is generally acknowledged that there is a very low rate of identification and treatment of mental disorders [21]. Prison inmates with mental disorders are in some cases incarcerated with the general prison population and little effort is made to provide mental health care [24].

Unfortunately, there is currently very little research on these topics in Nigeria, the seventh most populous country in the world and the largest in sub-Saharan Africa. What research does exist is dominated by the relatively economically prosperous southern parts of the country. This paper assesses the current situation at the Benue State Makurdi Medium Security Correctional facility in Nigeria’s North Central region.

Our aim is to help inform the development of interventions and services for prison inmates with depression in the North Central region by first assessing the current situation at the Benue State Makurdi Medium Security Correctional Facility, from the perspectives of prison inmates themselves. Our objective is to carry out a cross-sectional descriptive study with a random sample of prisoners, focused on the following questions:

1. What are the social and economic characteristics of inmates at this correctional facility?
2. How did these inmates come to be in custody, and what has been their experience of the criminal justice system?
3. What are the psychosocial consequences of being remanded in custody for these inmates?
4. What is the prevalence of depression among these inmates?
5. How has the correctional facility responded (or failed to respond) to inmates’ mental health needs?

Findings from this study will be relevant not only to the correctional system in Benue State and in Nigeria more broadly, but also to other LMICs seeking to improve the mental health of inmates, in response to global calls for action [1].

## Methods

### Study design

This was a descriptive cross-sectional study carried out at Benue State Makurdi Medium Security Correctional Facility. Data were collected between August and September 2017.

### Setting and population

#### Makurdi Medium Security Correctional Facility

Makurdi Medium Security Correctional Facility was commissioned in 2001 under the oversight of the Controller of Prisons, Benue State Command. Facilities comprise an administrative block, records section, gate lodge, welfare section, industrial workshop and a medical unit, as well as a prison yard with some recreational facilities (such as a football pitch). While the original proposed inmate capacity was 240, the facility currently holds around 900, the majority of whom are awaiting trial [25].

The medical unit has one general duty doctor, a pharmacist, a clinical psychologist, two laboratory technicians, five nurses, two Community Health Extension Workers and two auxiliary nurses. There is no psychiatrist, psychiatric nurse, social worker or occupational therapist on staff. Psychotropic medications are not available in the facility. The correctional facility reports that psychosocial services available include general counseling, exercise and skill acquisition. Health talks are also given to inmates who attend the clinic. These services are provided by health center staff. Christian organizations that visit the facility offer prayers and administer anointing oil. No service user organization or self-help group is in existence at the facility. Some inmates with mental illnesses are referred on occasion to the psychiatric clinic of the Federal Medical Centre, Makurdi for evaluation, treatment and court reports.

### Sample

#### Sample size determination

Applying the Cochran formula for populations greater than 10,000 (z^2^pq/d^2^), with precision set at 5%, z = A constant at 95%, confidence interval = 1.96 and N = target population, and using a prevalence of 34%, the calculated sample size was inflated by 10% to account for any potential retrospective withdrawal of consent resulting in a total calculated sample size of 381 [26]. The prevalence estimate was based on a Nigerian study of the mental health of 100 inmates of a medium security prison [15].

### Eligibility criteria

Adult (age 18+) men and women were eligible for inclusion if they were inmates at the Benue State Makurdi Medium Security Correctional Facility at the time of the survey. Prisoners who did not give written informed consent to participate in the study were excluded.

### Selection

From the correctional facility record, a register containing the names of all the eligible inmates was created. There were 902 names on the register. These were coded from 001 to 902. On pieces of paper, numbers ranging from 001 to 902 were written, and were then folded and shaken. The first 381 numbers were picked through simple random sampling by replacement. The register was restricted only to the Principal Investigator and utilized solely for the recruitment and interview process. At the end of the exercise, the master list was shredded. These steps were taken to guarantee respondent anonymity.

### Instruments

The study instruments include a structured questionnaire and Patient Health Questionnaire 9 (PHQ-9). Instruments were translated from English into Tiv and then back-translated into English. The original English versions were then compared against the back-translated versions to check for accuracy. Minor differences were resolved by the bilingual translators to produce the final consensus versions that were used in this study.

#### Structured questionnaire

The structured questionnaire was adapted from a prison questionnaire previously developed for use in Nigeria [17]. It is divided into four sections: (1) socio-demographic data, (2) forensic data, (3) experiences of prison and impact, and (4) previous medical history.

In, “Background” section, socio-demographic data included questions on age, sex, educational status, employment status, income, religious affiliation, tribe, marital status, duration of marriage prior to imprisonment and number of children. “Methods” section covered forensic data, including charge (s) against the participants, repeat offending (previous arrest and imprisonment, charges, frequency and reasons for repeat offending), time in prison and trial status (convicted or awaiting trial). For those convicted, we also asked about the length of sentence. For those awaiting trial, we asked about the plea, number of times in court, representation, self-assessment of the quality of representation and frequency of adjournments. “Results” section covered self-reported experience of the prison, in terms of quality of food, accommodation, clothing, general health condition, visitors, recreational facilities, educational rehabilitation, occupational rehabilitation, and freedom of worship. It also covered the impact of stay on family, occupation, relationships, the religious life of inmates and any history of mental illness. “Discussion” section covered prior medical and psychiatric history,

#### Patient Health Questionnaire 9

This is a short nine-item screening tool for symptoms of depression, which has been used in previous studies of non-specialist settings in Nigeria [27, 28]. Depression is indicated if five or more of the nine symptom criteria have been present at least “more than half the days” in the past 2 weeks, and one of the symptoms is depressed mood or anhedonia [29]. For the purposes of this study, those with scores ranging between zero and four were considered not to have depression.

### Procedure

The selected inmates were approached by trained research assistants to explain the study and obtain consent during periods of recreation in the yard. It was explained that participation in the study is voluntary, non-participation will not affect the inmate in any way, and they are free to opt out of the study at any time. The study instruments were later administered to the selected inmates on a one-to-one basis in a private room by research assistants. The four research assistants were all conversant in both English and Tiv. Participants who could speak English were interviewed using the English version of the instruments, while others were interviewed using the Tiv version.

### Analysis

The data generated were analyzed using the Statistical Package for the Social Sciences (SPSS) version 16 software. Frequencies and cross-tabulation of variables were generated to check for data entry errors and missing values. Descriptive statistics were calculated, including frequencies and percentages for categorical variables, and means with standard deviations for continuous variables. A post-hoc exploratory analysis was done. Chi-square test for categorical variables and t-test for continuous variables were used to test for association between depression and socio-demographic variables. It was believed that though the study was not powered for this, a post-hoc analysis would help in generating hypotheses for future research.

#### Ethics

Approval for the study was obtained from the University of Ibadan/University College Hospital Ethics and Research Committee. Necessary permissions and clearances were also obtained from the Prison Command Authorities. Only those who provided consent to be interviewed were recruited. The prison inmates were informed that they were completely free to refuse participation, though most welcomed it as an opportunity to contribute to the improvement of health facilities in the prison. The participants were given toiletries and soft beverages to thank them for their time. However, these were judged to be modest enough so as to avoid undue influence to the decision to participate.

## Results

### Social and economic characteristics of inmates

Table 1 shows the participants’ socio-demographic characteristics. The sample consisted of 381 participants. The mean age of participants was 27.95 ± 7.08 years with a majority, (n = 320, 84.0%) aged 15–34 years. Most were male (n = 364, 95.5%), and a large percentage (n = 242, 63.5%) had completed secondary education. The vast majority was Christian (n = 358, 94.0%), and Tiv (n = 251, 65.9%) was the dominant tribe.

**Table 1:** Socio-demographic characteristics

Variables	Frequency	Percent
Sex		
Male	364	95.5
Female	17	4.5
Mean age	27.95 ± 7.08	
Educational status		
None	22	5.8
Completed primary	68	17.8
Completed secondary	242	63.5
Completed tertiary	49	12.9
Religion		
Christianity	358	94.0
Islam	23	6.0
Tribe		
Tiv	250	65.9
Others	129	34.1
State of origin		
Benue	320	84.0
Other	61	16.0
Employment before arrest		
Yes	313	82.2
No	68	17.8
Occupation (n = 313)		
Civil servant	15	4.8
Self-employed	298	95.2
Average monthly income before arrest (in Naira) (n = 313)		
< 20,000	118	37.7
20,000–100,000	182	58.1
> 100,000	13	4.2
Mean income	39,301.02 ± 72,789.25	
Marital status		
Single	200	52.5
Married	163	42.8
Divorced/widowed	18	4.7
Family setting (n = 163) Family type, if married		
Monogamous	120	73.682.8
Polygamous	43	26.4
Number of children (n = 179)		
1–4	156	87.1
≥ 5	23	12.6

A high percentage (n = 313, 82.2%) was employed before the arrest; of these, 298 (95.2%) were self-employed. The overall mean income was N39, 301 (109 USD) with more than half, (n = 182, 58.2%) earning between N20, 000–N100, 000 (55.5–277.8 USD) monthly.

### Experience of the criminal justice system

Table 2 describes the participants’ experience with the criminal justice system. Some of the participants, (n = 182, 47.8%) had spent 5–20 days in police custody while 207 (54.3%) had spent > 150 days in prison. A high proportion, (n = 171, 44.9%) were charged with armed robbery. Almost all the participants, 377 (99.0%) were awaiting trial. Of those in detention, (n = 38, 10.1%) were yet to appear in court. Most, (n = 255, 67.6%) indicated that they have a lawyer. Most of the lawyers, (n = 209, 82.0%) were paid counsel. Slightly greater than half, (n = 185, 52.0%) were satisfied with their representation. The majority of the participants, (n = 260, 78.9%) indicated that their case had been adjourned on a number of occasions.

**Table 2:** Experience with the criminal justice system

Variables	Frequency	Percent (%)
Days in police custody		
< 5	26	6.8
5–20	182	47.8
> 20	173	45.4
Days in prison		
≤ 30	23	6.0
31–150	151	39.6
> 150	207	54.3
Post held in the cell		
Yes	71	18.6
No	310	81.4
Charge		
Armed robbery	171	44.9
Homicide	57	15.0
Theft	31	8.1
Criminal conspiracy	28	7.3
Kidnapping and terrorism	22	5.8
Others	72	18.8
Status		
Awaiting trial	377	99.0
Convicted	4	1.0
Plea		
Not guilty	345	91.5
Guilty	32	8.4
Appeared in court		
Yes	339	89.9
No	38	10.1
Number of times in court		
1–5	188	54.7
6–20	106	30.8
> 20	50	14.5
Represented by a lawyer		
Yes	255	67.6
No	122	32.4
Type of lawyer		
Paid counsel	209	82.0
JDPC	10	3.9
Legal aid	36	14.1
Ever been arrested before		
Yes	63	16.9
No	309	83.1
Number of times (n = 63)		
1	33	52.4
> 1	30	47.6
Charges during previous arrests (n = 63)		
Cultism	10	15.9
Theft	9	14.3
Fighting	11	17.5
Suspect in a crime	16	25.3
Others	17	27.0
Reason for reoffending (n = 63)		
No reason given	41	65.1
No job	7	11.1
Lack of capital to start a business	6	9.5
Forced back by a gang	4	6.3
No training for any other occupation	3	4.8
Crime more profitable	2	3.2

*JDPC* Justice, development and peace commission

Some of the participants, 63 (16.9%) had a history of previous arrests. Thirty-three (52.4%) of these had a history of a single previous arrest. Reasons reported for re-offending include joblessness (11.1% of re-offenders); lack of capital (9.5%); lack of skills (4.8%); coercion by a gang (6.3%), and perception of crime being more profitable (3.2%).

### Experience and social consequences of incarceration

Table 3 shows the experience and social consequences of incarceration. Most of the participants, (n = 331, 86.9%) rated the food as poor. Although, 244 (64.0%) indicated that accommodation was well given, 197 (51.7%) indicated that they slept on the bare floor. Over half, (n = 212, 55.6%) were satisfied with the clothing provided, but 190 (49.9%) had only one set of clothing and 156 (40.9%) had been wearing the same set for over 6 months. Few of the participants, (n = 44, 11.5%) participated in recreation and sports, and 367 (96.3%) had no access to educational rehabilitation. Most, (n = 352, 92.4%) reported that they had freedom of worship.

**Table 3:** The living situation in prison custody

Variables	Frequency	Percent (%)
Quality of food		
Poor	331	86.9
Good	50	13.1
Quality of accommodation		
Good	244	64.0
Poor	137	36.0
Where do you sleep		
On the bare floor	197	51.7
On a mattress on the floor	116	30.4
On a bed	68	17.8
Satisfaction with clothing		
Yes	212	55.6
No	169	44.4
Sets of clothing possessed		
1	190	49.9
> 1	191	50.1
How long have you been wearing them		
< 1 month	82	21.5
1–6 months	143	37.6
> 6 months	156	40.9
Recreation and sports		
Yes	44	11.5
No	337	88.5
Educational rehabilitation		
Yes	14	3.7
No	367	96.3
Freedom of worship		
Yes	352	92.4
No	29	7.6
Frequency of visitors from outside		
No visitors at all	97	25.5
Daily	23	6.0
Weekly	103	27.0
Monthly	153	40.2
Annually	5	1.3
*Those that have visited (multiple response)		
Parents	147	26.2
Other relatives	170	30.4
Spouse and children	91	16.2
Friends	87	15.5
Others	65	11.6
Currently attend workshop		
Yes	7	1.8
No	374	98.2
If No to statement above, why not?		
No Workshop organized	210	57.1
Only for convicts	102	27.7
Insufficient space	43	11.7
Don’t like the options	13	3.5

Many of the participants, (n = 91, 23.9%) never have visitors. For those who have visitors, parents 147 (26.2%) and other relatives 170 (30.4%) made up the majority of visitors.

Table 4 shows the perceived social consequences of imprisonment. The greatest impact of imprisonment on the family was social embarrassment (n = 276, 44.7%). The most painful loss suffered was the loss of a job (n = 191, 39.6%). Most, (n = 321, 84.3%) became more religious while a small but notable percentage (n = 60, 15.7%) reported they had lost their faith in God. About one third, (n = 120, 31.5%) anticipated difficulty with future employment.

**Table 4:** Perceived social consequences

Variables	Frequency	Percent (%)
Consequences on the family^a^		
Family embarrassed	276	44.7
Lost job	205	33.2
Children living with relatives	66	10.7
Spouse left	41	6.6
Children dropped out of school	29	4.7
Anticipated difficulty with future employment		
Yes	120	31.5
No	261	68.5
Religion		
Become more religious	321	84.3
Lost faith in God	60	15.7
Most painful loss^a^		
Job loss	191	39.6
Life ruined	125	25.9
Family and children neglected	117	24.3
Spousal abandonment	49	10.2
Emotional response to stay in prison^a^		
Become a better person	268	28.5
Seen that Government is unfair	233	24.7
Convinced nobody cares	179	19.0
Angry and bitter towards society	152	16.4
Learned to be smarter	110	11.7

^a^Multiple response

Emotional responses include the beliefs that their experience in prison made them better people (n = 268, 28.5%); that the government had been unfair to them (n = 233, 24.7%); that nobody cared (n = 179, 19%); and anger and bitterness toward society (n = 152, 16.4%).

### Mental health care structures

Table 5 shows mental health care structures available for inmates in prison. Eight (2.1%) of the participants had mental health problems before imprisonment. Six (1.6%) were on medication for a mental or emotional problem at the time of imprisonment. Twenty-seven (7.1%) of the participants were identified as suffering a mental disorder by prison health authorities. Of these, (n = 15, 3.9%) were diagnosed with depressive disorder. Only four (1.0%) of those identified with a mental disorder were placed on medication, while 26 (6.8%) had received professional counseling since admission in prison.

**Table 5:** Mental health care structures

Variables	Frequency	Percent (%)
Medical history taken on admission in prison		
Yes	67	17.6
No	314	82.4
Mental health problem prior to incarceration		
Yes	8	2.1
No	373	97.9
On treatment for mental or emotional problem at the time of incarceration		
Yes	6	1.6
No	375	98.4
Current mental health problem		
Yes	27	7.1
No	354	92.9
Diagnosed with depression by prison authorities		
Yes	15	3.9
No	366	96.1
Depressive disorder using PHQ9		
Not depressed	237	62.2
Depressed	144	37.8
On medication for a mental or emotional problem since incarceration		
Yes	4	1.0
No	377	99.0
Counseling by a trained professional since admission in prison		
Yes	26	6.8
No	355	93.2
Satisfaction with Healthcare provided		
Yes	183	48.0
No	198	52.0

Over half, (n = 198, 52.0%), of the participants were dissatisfied with the prison health care. Using PHQ9, 144 (37.8%) of the participants met the criteria for depression.

### Exploratory analysis

Table 6 shows association between depression and participant characteristics. There was a significantly greater rate of depression among those who were employed before arrest (39.9%) compared to 27.9% among those who were not employed (χ^2^ = 3.42, p = 0.04). There was a significantly greater rate of depression among those earned < 20,000 Naira average monthly income (48.3%) compared to 33.1% among those who earned > 20,000 Naira (χ^2^ = 8.03, p = 0.003). There was a significantly greater rate of depression among those who were dissatisfied with the healthcare provided in prison (44.4%) compared to 30.6% among those who were satisfied (χ^2^ = 7.75, p = 0.006). There was a significantly greater rate of depression among those who had spent 31–150 days in prison (48.3%) compared to 34.8% and 30.4% among those who had spent ≤ 30 days and > 150 days respectively (χ^2^ = 12.01, p = 0.002).

**Table 6:** Association of participant characteristics and depression

Variable	Depression status		Test statistic	P-value
	Not depressed n (%)	Depressed n (%)		
Gender				
Male	230 (63.2)	134 (36.8)	χ^2^ = 3.35	0.07
Female	7 (41.2)	10 (58.8)		
Age (in years)				
15–34	198 (61.9)	122 (38.1)	χ^2^ = 0.09	0.76
≥ 35	39 (63.9)	22 (36.1)		
Mean age ± SD	28.03 ± 7.53	27.82 ± 6.28	t = 0.28	0.78
Educational status				
Uneducated	15 (68.2)	7 (31.9)	χ^2^ = 0.36	0.55
Educated	222 (61.8)	137 (38.2)		
Religion				
Islam	14 (60.9)	9 (39.1)	χ^2^ = 0.02	0.53
Christianity	223 (62.3)	135 (37.7)		
Tribe				
Tiv	160 (63.7)	91 (36.3)	χ^2^ = 0. 74	0.39
Others	77 (59.2)	53 (40.8)		
State of origin				
Indigene (Benue)	201 (62.8)	119 (37.2)	χ^2^ = 0. 31	0.58
Non-indigene	36 (59.0)	25 (41.0)		
Employment before arrest				
Yes	188 (60.1)	125 (39.9)	χ^2^ = 3.42	0.04*
No	49 (72.1)	19 (27.9)		
Occupation				
Civil servant	9 (60.0)	6 (40.0)	χ^2^ = 0.32	0.53
Self employed	179 (60.1)	119 (39.9)		
Mean monthly income (Naira)	37,871 ± 47,070	41,452 ± 99,910	F = 0.18	0.67
Monthly income (in Naira)				
< 20,000	61 (70.9)	57 (48.3)	χ^2^ = 8.03	0.003*
> 20,000	176 (66.9)	87 (33.1)		
Marital status				
Single	142 (65.1)	76 (34.9)	χ^2^ = 1.86	0.10
Married	95 (58.3)	68 (41.7)		
Days in police custody (days)				
< 15	101 (56.4)	75 (42.6)	χ^2^ = 3.23	0.45
> 15	136 (66.3)	69 (33.7)		
Mean no. days in custody	27.6 ± 34.11	28.20 ± 53.6	t = 0.02	0.89
Days in prison				
≤ 30	15 (65.2)	8 (34.8)	χ^2^ = 12.01	0.002*
31–150	78 (51.7)	73 (48.3)		
> 150	144 (69.6)	63 (30.4)		
Mean no. days in prison	431.03 ± 590.03	322.37 ± 402.23	t = 3.80	0.05
Appeared in court				
Yes	212 (62.5)	127 (37.5)	χ^2^ = 0.01	0.54
No	24 (63.2)	14 (36.8)		
Number of times in court				
1–5	108 (57.4)	80 (42.6)	χ^2^ = 5.12	0.06
6–20	74 (69.8)	32 (30.2)		
> 20	34 (68.0)	16 (32.0)		
Represented by a lawyer				
Yes	162 (63.5)	93 (36.5)	χ^2^ = 0.29	0.33
No	74 (60.7)	48 (39.3)		
Satisfaction with Healthcare provided				
Yes	127 (69.4)	56 (30.6)	χ^2^ = 7.75	0.006*
No	110 (55.6)	88 (44.4)		
Quality of accommodation				
Good	158 (64.8)	86 (35.2)	χ^2^ = 1.87	0.10
Poor	79 (57.7)	58 (42.3)		

^*^significant

## Discussion

This paper assessed the mental health situation in a prison facility in North Central Nigeria, with a focus on depression. Striking findings include: (1) the high prevalence of depression in this prison inmate population; (2) the poor rate of identification of depression (by correctional facility authorities as well as by prison inmates themselves); (3) lack of treatment (particularly psychopharmacological) for mental disorders generally; (4) moderate levels of satisfaction with correctional facility healthcare; and (5) extremely high numbers of prison inmates who had not been formally convicted. Our exploratory analysis further suggests that in addition to socio-demographic characteristics that precede imprisonment, experiences of imprisonment and the prison environment may be associated with depression.

The prevalence of depression (37.8%) as measured via the PHQ-9 screening tool is higher than the pooled prevalence of mood disorders in the African region (22%) [5]. It is similar to that reported by Armiya’u et al. [9, 10] in Jos (30.8%), but much higher than the reported prevalence of depression of 20.8% in a similar prison population from Ibadan [17]. These disparities may point to geographical differences, as Jos is also based in the North Central Region, while Ibadan is in the more prosperous southwest of Nigeria.

That our findings regarding prevalence are concurrent with those of Armiya’u et al. is of interest, given their use of an arguably more rigorous two-stage process for identification, plus a different (and much longer) screening tool (GHQ-28) than the PHQ-9. If both studies have indeed captured the true prevalence of depression in their respective locations, this would suggest not only that prisoners’ mental health is perhaps similar between these two states in the North Central region, but also that screening by PHQ-9 could be an efficient way to identify prisoners with depression in these contexts. However, further research is needed to explore both of these points.

We found that only a small number of those with a mental disorder were identified by the correctional facility authorities, and the number of prison inmates who screened positive for depression was much higher than the number identified as having a mental disorder by the prison authorities. It is possible that both prison authorities and prisoners do not recognise symptoms of depression as those of a mental disorder. It could also be that in an environment with limited access to mental health care, there is no motivation to identify prison inmates as having depression, which will likely go untreated anyway. However, studies from high-income countries also report that psychotic disorders are more easily identified by correctional facility authorities than mood disorders and recommend routine screening to improve detection rates [30, 31].

Only a small proportion of prison inmates identified as having a mental disorder were offered psychotropic medication. This is similar to findings from high-income countries regarding treatment of prison inmates with mental disorders. For example, a 2014 analysis of data from over 18,000 American prison inmates found that treatment was disrupted upon admission for the majority of prison inmates with a history of mental disorders [31]. Only a small proportion of prison inmates with a lifetime diagnosis of a mental disorder continue with their treatment upon admission in prison [31]. This has been attributed to a lack of appropriately skilled human resources, especially psychologists and psychiatrists, to properly diagnose and treat mental disorders [32].

Given the poor access to mental health care and the poor conditions generally reported by inmates, it is rather surprising that almost half (48%) reported they were satisfied with the medical treatment at the prison. This could perhaps be a reflection of the extreme poverty and poor access to care that many inmates experienced prior to imprisonment, or else habituation to the conditions of the prison system. Prisoners’ expectations may be very low, resulting in a certain degree of satisfaction with whatever goods or services are provided by the correctional facility authorities. However, results from our exploratory analysis suggesting that those with depression were less likely to be satisfied with the correctional facility’s health service could also be a reflection on the relative paucity of mental health care compared to physical health care at this facility.

Very concerning is that 99% of participants in our sample had not been convicted of a crime. Most were still undergoing trial while some had not yet appeared in court at all. Even among those who had been to court, many had appeared several times with representation and still had their cases adjourned. This is a major problem in the criminal justice system in Nigeria, leading to congestion in correctional facilities country-wide, and there is little likelihood that this situation will change in the near future [33]. This situation in which the majority of prison inmates were awaiting trial, was cited as the motivation for a study carried out at the Agodi Medium Security Prison of Ibadan by Abdulmalik, Adedokun, and Baiyewu [17]. Their study exclusively interviewed the inmates awaiting trial, who accounted for 91.6% of the prison population, as at the time of the study in 2013 [17]. It is also a common problem in the African region more broadly. A systematic review of 80 studies on the mental health of prisoners in sub-Saharan Africa found that in 36% of studies, the majority of participants had not been convicted of a crime [5]. This is a major source of psychosocial stress for prison inmates which could be contributing to high rates of depression, particularly given the association between depression and prolonged prison time indicated by our exploratory analysis.

Lack of access to a speedy trial also has implications for the services available to prison inmates. In our study, most prison inmates were not offered educational or occupational rehabilitation because in this low-resource environment, the limited facilities available are reserved for those who have already been convicted. This undermines the role of prisons as rehabilitation institutions with the ultimate goal of re-orientating and reforming inmates [33, 34]. Under Cap. 366 Laws of the Federation of Nigeria 1990 which governs the prison system, the correctional facility is expected to prepare inmates for eventual reintegration into society as law-abiding citizens (34). This cannot be achieved under poor conditions and without access to speedy trials.

Results of our exploratory analysis further reinforce the well-established relationship between poverty and common mental disorder (depression and anxiety) in LMICs [35]. Having an average monthly income lower than the minimum wage in Nigeria was associated with depression. Having some form of employment before arrest (and presumably either losing it or having it threatened as a result of imprisonment) was also associated with depression. A meta-analysis of prospective observational studies from mainly high-income countries has established that job insecurity and unemployment both represent significant risk factors for depression [36]

## Limitations

This was a cross-sectional study conducted among prison inmates in Benue state. Due to demographic differences and conditions in different prisons in Nigeria, the findings may not be generalized to the entire nation. Social desirability and recall bias cannot be ruled out, particularly given that the information provided by prison inmates about conditions in the correctional facility was through self-report.

Although it has been validated for use in non-specialist settings in Nigeria, to the best of our knowledge, PHQ-9 has not been validated for the screening of depression in Nigerian correctional facilities, specifically. Further, PHQ-9 does not screen for other mental disorders that are found in prison populations, such as psychotic disorders. The true prevalence of mental disorders in this population is likely much higher than the prevalence of depression alone [3]. We also recognize that some of our research questions are difficult to address using quantitative methods alone, and could benefit from further exploration and triangulation using qualitative methods. Due to the absence of routine screening, it was not possible to determine the proportion of inmates who meet the criteria for depressive disorder at the point of entry into the facility. This should be addressed in future research, in order to better understand the direction of causality between depression and experiences of imprisonment. A longitudinal study design would have been better equipped to investigate at what point those awaiting trial begin to manifest symptoms of depression and psychological distress.

## Recommendations

Despite the above limitations, this study does add to the existing evidence that the prevalence of mental conditions is high in Nigerian correctional facilities—northern as well as southern institutions [5–7, 9, 10]. This is unlikely to change while the country struggles to ensure access to fair and speedy trials, resulting in congestion and poor living conditions in its correctional facilities. Based on our findings, we recommend that efforts should be made to expedite trials in the correctional facility in Benue State and across the country to decongest these facilities. Not only is overcrowding an environmental stressor that may contribute to poor mental health, but the experience of prolonged detention without trial takes its own psychological toll [3, 4, 18, 19]. It is also possible that innocent detainees may be unnecessarily exposed to the various risk factors associated with imprisonment over a long period of time while awaiting trial [13].

We also recommend that prisons do more to identify and care for people with mental health conditions, potentially reducing the risk of reoffending and therefore relieving some of the pressure on the justice system. We suggest adopting standard screening procedures for under-detected mental disorders like depression, and ensuring that mental health services in correctional facilities are properly resourced. Many screening tools can be administered by trained lay people such as motivated prison staff [37]. Screening should ideally take place first at the point of admission into the facility and then at other routinely designated times during their stay to detect changes. In order to be effective, screening would need to be supported through appropriate supervision, monitoring, and functioning referral pathways for those who screen positive [32]. In the absence of specialist mental health staff, Makurdi Medium Security correctional facility should consider training its non-specialist medical staff in the World Health Organisation’s mental health Gap Action Programme Intervention Guide (mhGAP-IG) [38]. This would also require strengthening referral pathways to ensure that those with complex cases receive necessary specialist care from one of Makurdi’s tertiary facilities. Given the mhGAP-IG’s emphasis on psychotherapy for mood disorders and the prison’s difficulties in providing psycho-pharmaceuticals, it could also consider training staff to provide manualized psychotherapy. A small controlled study in Enugu State, for example, has shown that a group-focused cognitive-behavioural coaching programme can reduce depression symptoms among inmates [39]. However, this is an under-researched area, and more studies are needed to determine which therapies are most cost-effective to deliver in LMIC correctional facility populations. This could be a topic for further study at Makurdi Medium Security Correctional Facility.

## Conclusion

Our paper reinforces calls by previous researchers to improve living conditions in correctional facilities in Nigeria, in line with international human rights instruments [1]. It adds to existing evidence of poor conditions experienced by prison inmates with new evidence suggesting very high rates of depression and unmet need for mental health care in this correctional facility [31]. Poor mental health increases risk of reoffending and other negative outcomes, such as suicide, violence and victimization in correctional facility [3, 4, 18, 19]. Improving mental health among prisoners in Nigeria will require not only better detection and treatment, but also structural and environmental changes to reduce exposure to known risk factors—in particular, prolonged detention without trial.

## Data Availability

The data set used and analyzed during the study is available from CBM on reasonable request.
